# Evaluating the Effects of Population Management on a Herbivore Grazing Conflict

**DOI:** 10.1371/journal.pone.0056287

**Published:** 2013-02-06

**Authors:** Kevin A. Wood, Richard A. Stillman, Francis Daunt, Matthew T. O’Hare

**Affiliations:** 1 Centre for Ecology and Hydrology, Bush Estate, Penicuik, Edinburgh, Midlothian, United Kingdom; 2 School of Applied Sciences, Bournemouth University, Talbot Campus, Poole, Dorset, United Kingdom; University of Otago, New Zealand

## Abstract

Abundant herbivores can damage plants and so cause conflict with conservation, agricultural, and fisheries interests. Management of herbivore populations is a potential tool to alleviate such conflicts but may raise concerns about the economic and ethical costs of implementation, especially if the herbivores are ‘charismatic’ and popular with the public. Thus it is critical to evaluate the probability of achieving the desired ecological outcomes before proceeding to a field trial. Here we assessed the potential for population control to resolve a conflict of non-breeding swans grazing in river catchments. We used a mathematical model to evaluate the consequences of three population management strategies; (a) reductions in reproductive success, (b) removal of individuals, and (c) reduced reproductive success and removal of individuals combined. This model gave accurate projections of historical changes in population size for the two rivers for which data were available. Our model projected that the River Frome swan population would increase by 54%, from 257 to 397 individuals, over 17 years in the absence of population control. Removal of ≥60% of non-breeding individuals each year was projected to reduce the catchment population below the level for which grazing conflicts have been previously reported. Reducing reproductive success, even to 0 eggs per nest, failed to achieve the population reduction required. High adult and juvenile survival probabilities (>0.7) and immigration from outside of the catchment limited the effects of management on population size. Given the high, sustained effort required, population control does not represent an effective management option for preventing the grazing conflicts in river catchments. Our study highlights the need to evaluate the effects of different management techniques, both alone and in combination, prior to field trials. Population models, such as the one presented here, can provide a cost-effective and ethical means of such evaluations.

## Introduction

How to manage species and environments for the benefits of conservation, economic, and human well-being objectives is a central challenge facing ecologists [1]. Such management can often involve population control where humans intervene to reduce population size in a range of species, for example to eradicate invasive species and limit the population of agricultural pests [2], [3], [4]. Population control typically focuses on reducing the number of individuals through translocation [5], [6] or culling [7], [8], or on reducing reproductive output through fertility control [4], [6], [9] or, in birds, destroying eggs [2], [10]. Selecting appropriate methods of population control requires consideration of the ecological, economic, and ethical consequences of control [3], [6], [11]. Aside from the financial costs of management, which can be great, the manipulation of animal populations can also be an emotive issue, particularly where lethal methods are used or target species are charismatic [12], [13], [14], [15]. Thus managers must ensure that methods of population control are likely to achieve the desired ecological outcomes, are cost effective, and are conducted in the most ethical manner possible.

Population control measures on vertebrate herbivores typically focuses on reducing population size by (i) reducing reproductive success to reduce recruitment into the population, or (ii) removing individuals to reduce population size directly [4], [6], [7], [8], [16]. Previous research has demonstrated that single applications of a population control (i.e. carried out in one year only) on established, open populations of long-lived animals capable of rapid dispersal is seldom effective, with rapid (<5 years) recovery from any reductions [7], [17], [18]. Population control measures in long-lived species may also be associated with a lag time between management and the resulting change in population size [19], [20]. Thus the effects of repeated annual population control sustained and monitored over a biologically meaningful period of time should be tested [21]. In general, strategies which minimise both the total number of individuals which must be killed, and the duration of time over which culling must be carried out, are considered to be more ethically palatable to the public and stakeholder groups [3], [14].

Large vertebrate herbivores can damage vegetation of ecological and socioeconomic value through consumption, trampling and altered nutrient concentrations [22], [23], [24], [25]. Such damage may require management to alleviate the effects of herbivory and protect the plants and other organisms that depend on such plants [3], [4], [6], [16]. Mute swans (*Cygnus olor* Gmelin, 1789) are charismatic herbivores known to reduce plant abundances in both aquatic and terrestrial habitats [26], [27], [28], [29]. Recent studies have reported that swan flocks can cause substantial reductions in aquatic plant abundance in the chalk rivers of southern England [28], [29], [30]; such plants are protected under the EU Habitats and Species Directive (92/43/EEC) due to the abundant and diverse biota that are supported. Swan herbivory also occurs in pasture fields adjacent to chalk rivers; a mean pasture grass yield loss of 11.4% in fields grazed by flocks of swans has been reported, which increased livestock feeding costs for the farmers affected [26]. Thus a conflict exists in chalk river catchments between a protected charismatic herbivore species and the protected, high-value plants that are damaged.

Mute swans are protected under the EU Wild Birds Directive (2009/147/EEC), implemented in the UK through the Wildlife and Countryside Act (1981), making it illegal to capture, kill or injure swans, or to disturb or damage swan nests or eggs [31]. However, management which removes adults or eggs could be allowed under licence where substantial damage to agricultural, fisheries and conservation interests was demonstrated. Reducing the number of swans in areas of conflict through population management has been suggested as a means to alleviate swan grazing conflicts [16], [17]. However, recent culls of mute swan and black swan (*Cygnus atratus* Latham, 1790) populations in the United States and New Zealand respectively faced widespread public opposition, legal challenges, and damaged relations between different stakeholder groups, in particular government wildlife officials, landowners and animal welfare groups [11], [14]. A major point of contention between these stakeholders was whether the proposed methods of population control were the most effective means of alleviating herbivore damage [12], [14]. In general, the manipulation of clutch sizes is considered the least ethically controversial method, and combined removal of birds and clutch control is likely to attract most opposition. Therefore the suite of available population management methods represent a wide range in terms of costs, ethics, and crucially, ecological outcomes. Thus evaluations of the probabilities of different methods of population control achieving their desired ecological outcomes, in a cost-effective and ethical manner, should be conducted prior to field trials to minimise the rancour experienced during the schemes in the US and New Zealand.

Mathematical models allow the projection and evaluation of the consequences of management decisions on species abundances and distributions [7], [32], [33], [34], [35]. For example, such models have been used previously to evaluate the effectiveness of different techniques, such as culling and egg destruction, in reducing population sizes of nuisance waterfowl species [3]. In this paper we developed a mathematical model to evaluate the consequences of different management strategies on a swan population at the scale of a river catchment. We built on previous research by constructing and testing a model of a mute swan population in a chalk river catchment [3], [16]. A previous study used a small-scale (16.9 km river length) model to project the population-level outcomes of reducing reproductive output through the destruction of eggs in a UK chalk river swan population [16]. However, this small-scale may preclude accurate projections of the numerical responses of swan populations to management; swans exhibit seasonal movements between different habitats within a river catchment, suggesting that a more appropriate scale would be the catchment-scale [36]. Another population model was used to inform the eradication of an invasive mute swan population in North America [3]. However, the potential differences in demography between a rapidly-expanding invasive population and a population within its natural range, as well as the different management objectives, mean that a new model is required to test the range of proposed management strategies for chalk river swan populations. Breeding swans are highly territorial, excluding other individuals from a given area, which is a key factor regulating mute swan population dynamics [17], [36], [37]; however, territoriality has not been incorporated into previous swan population models. Thus in our model we allow the number of breeding pairs to be regulated in part by territory availability. Swans are known to immigrate into chalk rivers from outside the catchment, although the number of immigrants likely varies considerably between different chalk rivers [16], [17], [36]. To make general projections across different chalk river catchments it is crucial to understand how population-level responses to management vary with different levels of immigration.

In this study we first tested the ability of an age structured population model, incorporating territory availability, to generate accurate projections (sensu [33]) of historical changes in population size for two different chalk river swan populations. We then used this model to project the population-level responses over time to (a) the removal of adult or juvenile swans, simulating the effects of translocation or culling, (b) reductions in reproductive output, simulating the effects of fertility control or egg destruction, and (c) the combined effects of (a) and (b). Thus our study represents the most comprehensive assessment to date of the potential of different methods of population management to alleviate swan grazing conflicts in rivers.

## Methods

### Study System

The River Frome (Dorset, UK) is a shallow (<1.5 m depth), mesotrophic chalk river that flows through a catchment dominated by mixed pastoral and arable agriculture [38]. Our model was constructed for the 68.5 km length of the River Frome catchment described by [29]. The catchment has a mean population at the end of the breeding season of 257 swans [36]. Complaints of grazing damage in the River Frome catchment from stakeholder groups began after the year 1996 [36]. Thus reducing the swan population to pre-1996 levels may alleviate the grazing conflict. Whilst historical data for the entire River Frome catchment is lacking, surveys of the lower 19.4 km of the catchment in 1995 as part of a national monitoring programme reported a population size of 73 individuals [39]. Based on the 16 recent total catchment surveys reported in [29], [36], the lower 19.4 km accounts for a mean (±95% CI) of 47±5% of the total catchment population. Thus in 1995 the total catchment population was estimated at 155±14 individuals, equivalent to a population density of 2.3 ind. km^−1^ of river. This estimate seems sensible as the two chalk river systems for which grazing conflicts are known have densities which exceed this; the River Wylye density is 5.5 ind. km^−1^ and the current River Frome density is 3.8 ind. km^−1^ [17], [36].

### Population Model

**Figure 1:**
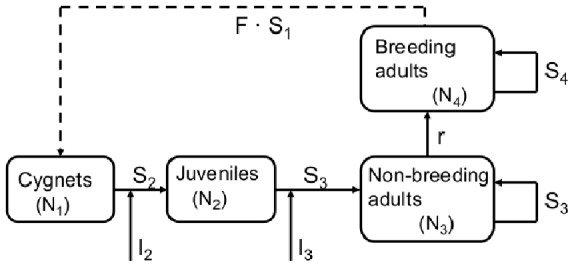
A conceptual description of the population model, indicating how the numbers of cygnets (N_1_), juveniles (N_2_), non-breeding adults (N_3_) and breeding adults (N_4_) were calculated. F and S refer to fecundity, and survival, respectively, whilst r refers to the recruitment of non-breeding adults into the breeding subpopulation.



We constructed an age-structured population model with a twelve month time step [33] (**Figure 1**). The models consisted of four life stages of swan; cygnets (N_1_), juveniles (N_2_), non-breeding adults (N_3_) and breeding adults (N_4_). Adult swans were further subdivided into breeding and non-breeding adults based on territory availability; adults were defined as breeding if they were randomly allocated one of the fixed number of territories (T).

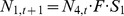


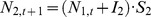


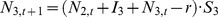


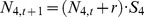



All model parameters were derived from data presented in studies of chalk river swan populations (**Table 1**). The terms F, S and I refer to fecundity, survival rate and number of immigrants respectively. The term r refers to recruitment of non-breeding adults into the breeding adult subpopulation.
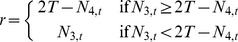



**Table 1:** Population model parameter mean values and standard deviations; see text for derivation.

Parameter	Mean	SD	Reference
Initial number of adults (N_3+4_)	147	±2.83	[36]
Initial number of juveniles (N_2_)	52	±31.11	[36]
Initial number of cygnets (N_1_)	58	±9.19	[36]
Number of breeding territories (T)	38	±9.24	[17], [36]
Number of eggs per breeding adult (F)	2.20	±2.80	[36]
Breeding adult survival rate (S_4_)	0.90	±0.11	[16] *
Non-breeding adult survival rate (S_3_)	0.71	±0.23	[16]
Juvenile survival rate (S_2_)	0.73	±0.25	[16]
Cygnet survival rate (S_1_)	0.37	±0.36	[36]
Number of adult immigrants (I_3_)	43.2	±17.81	[16], [36]
Number of juvenile immigrants (I_2_)	6.9	±2.74	[16], [36]

* The upper limit for breeding adult survival rate was set to 1.00 in the sensitivity analysis, as survival cannot exceed this threshold.

We estimated T as 38±9.24, after the mean densities of breeding pairs per length of chalk river reported by [17], [36]. We based the initial demographic distribution (*i.e.* relative numbers of adults, juveniles and cygnets) on the mean (± SD) September counts given in [36]. The model time step ran from October to September, as September is typically the month in which cygnets fledge and become juveniles, and begin to leave their natal territory [36], [40]. Juvenile and adult survival rates, as estimated by [16], represented the probability of an individual not leaving the study area either through death or emigration, and as such represent ‘apparent survival’. No cygnet immigration was permitted in the models as swans do not typically leave their natal territory until at least seven months old, *i.e.* until they have become juveniles [40]. In chalk river swan populations immigration occurs as birds fledged on waterbodies away from the river join the flocks on the river or adjacent pasture fields [17], [36]. Thus the number of immigrants was assumed to be proportional to the length of the river, *i.e.* a longer section of river should receive more immigrants as it has a larger surrounding area to supply such immigrants. The mean annual numbers of juvenile and adult immigrants to a 16.9 km length of chalk river have been estimated as 1.66±0.73 SD and 10.58±4.44 SD respectively [16], indicating 0.10±0.04 juveniles and 0.63±0.26 adult immigrants per km of river length. Thus our 68.5 km river length was assumed to receive 6.9±2.7 juvenile and 43.2±17.8 adult immigrants per year. These values are within the range of immigrant numbers reported for populations of mute swans in Britain [40], [41], [42]. Such values were also within the seasonal variation in the River Frome swan population size [36].

Even deterministic models with relatively simple structures can yield complicated population dynamics [43], [44]; thus it is critical to allow the model to make projections for a sufficient time for model properties to emerge and for an equilibrium population size, if one exists, to be identified. The models ran over a 50 year period, which allowed time for model properties to emerge whilst also being a time scale relevant to managers [3].

### Model Validation

To test the accuracy of the projections of our model, we compared model projections with historical population data for the swan populations of a 34.8 km section of the River Wylye [17] and a 19.4 km section of the River Frome [39]. Historical data for both populations spanned 16-year periods, although the time series for the River Frome was incomplete. We assessed the accuracy of our model by calculating the projected population size as a percentage of the observed population size for each year in the simulation (River Wylye: 1978 to 1993; River Frome: 1992 to 2007). From these yearly accuracy values we could calculate a mean accuracy value for the whole simulation.

### Management Strategies Tested

We tested three management strategies; (a) the effects of manipulating reproductive success, (b) the effects of removing a given percentage of the non-breeding subpopulation, and (c) the combined effects of (a) and (b), on the projected swan population size. The non-breeding subpopulation was targeted as grazing conflicts have been reported for these, but not breeding, individuals and thus removal of breeding birds is likely to prove unacceptable to stakeholders [12], [17], [28]. We used the number of eggs per nest as our estimate of reproductive success. Clutch sizes between 0 and 4 eggs per nest were tested, covering the full range of possible reductions in clutch sizes below the 4.4 reported for the River Frome [36]. The non-breeding subpopulation was the sum of the non-breeding adults and juveniles; we tested values of non-breeder removal at 10% increments between 0 and 100% inclusive in order to test the full range of options.

### Sensitivity Analysis

We assessed the sensitivity of the projected population size to changes in the mean value of each parameter using the one-at-a-time method of local sensitivity analysis [45]. To examine which parameters had the greatest relative effect on model projections, we subjected each parameter in turn to (a) an increase of 5% and (b) a decrease of 5%. As we were interested in the population outcomes projected by our model we compared the effects of parameter value changes on the projected stable population size. We defined the stable population size as the mean population size between years 36 and 50 of the simulations.

## Results

### Model Validation

**Figure 2:**
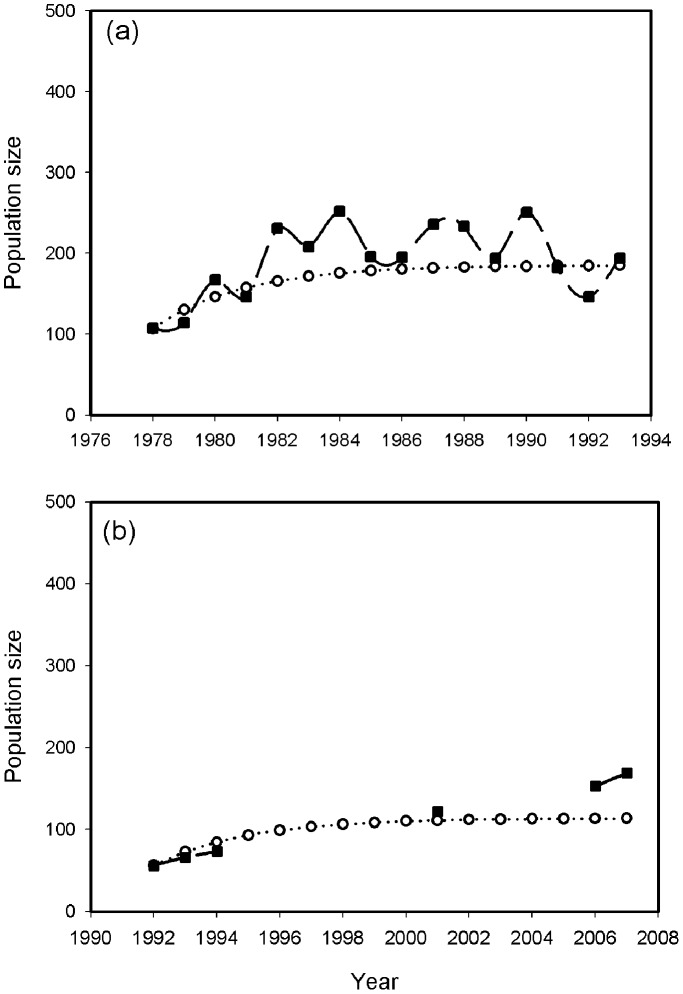
Comparisons between observed historical data (solid squares) and model projections (open circles) of the swan populations in (a) the River Wylye and (b) the River Frome.

The population sizes projected by our model, as a mean percentage of the observed population sizes, were 91% (range 72–126%) for the River Wylye population (**Figure 2a**). For the River Frome the model projections were 109% (range 82–126%) of the observed population (**Figure 2b**).

### Population Management

**Figure 3:**
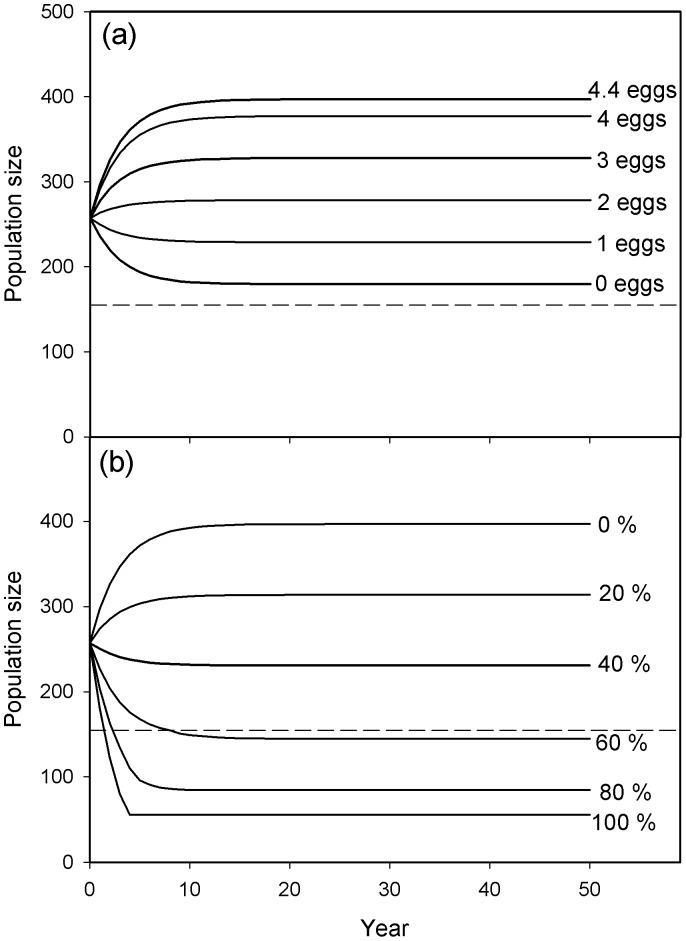
The projected population sizes when subject to sustained population management over a 50 year period. (a) Annual reductions in clutch size from 4.4 to 4, 3, 2, 1 or 0 eggs per nest. (b) Annual removal of 100, 80, 60, 40, 20 or 0% of the non-breeding individuals within the catchment. The dashed line represents the target threshold of 155 individuals.

The model projected a 54% increase in population size, from 257 to 397 individuals, within 17 years if no management action were taken (**Figure 3**). Our use of the mean population size between years 36 to 50 within a simulation as our estimate of the stable population size was supported as no variance in population size was detected during this 15-year period. Following the start of clutch reductions, population sizes were projected to take between 10 and 19 years (mean = 15) to reach a stable size, *i.e.* a population size which did not vary thereafter (**Figure 3a**). Once the yearly removals of non-breeding individuals had begun, population sizes were projected to reach a stable level within 4 to 17 years (mean = 12) (**Figure 3b**).

As expected, reducing the number of eggs per nest below the current mean value of 4.4 resulted in lower population sizes; greater reductions in clutch size resulted in lower population sizes (**Figure 3a**). However, no reduction in the number of eggs per nest was projected to yield a population size below our target threshold of 155 individuals. Reducing clutch size to 0 was projected to cause a decline in population size from 257 to 179 individuals within 19 years, stabilising at this lower level thereafter.

Our model projected that the sustained removal of ≥60% of non-breeding birds each year would achieve a stable population below the target threshold of 155 swans (**Figure 3b**). The greatest population reduction was achieved by removing 100% of non-breeding individuals annually, which was projected to reduce the population size from 257 to 56 individuals in 4 years.

**Figure 4:**
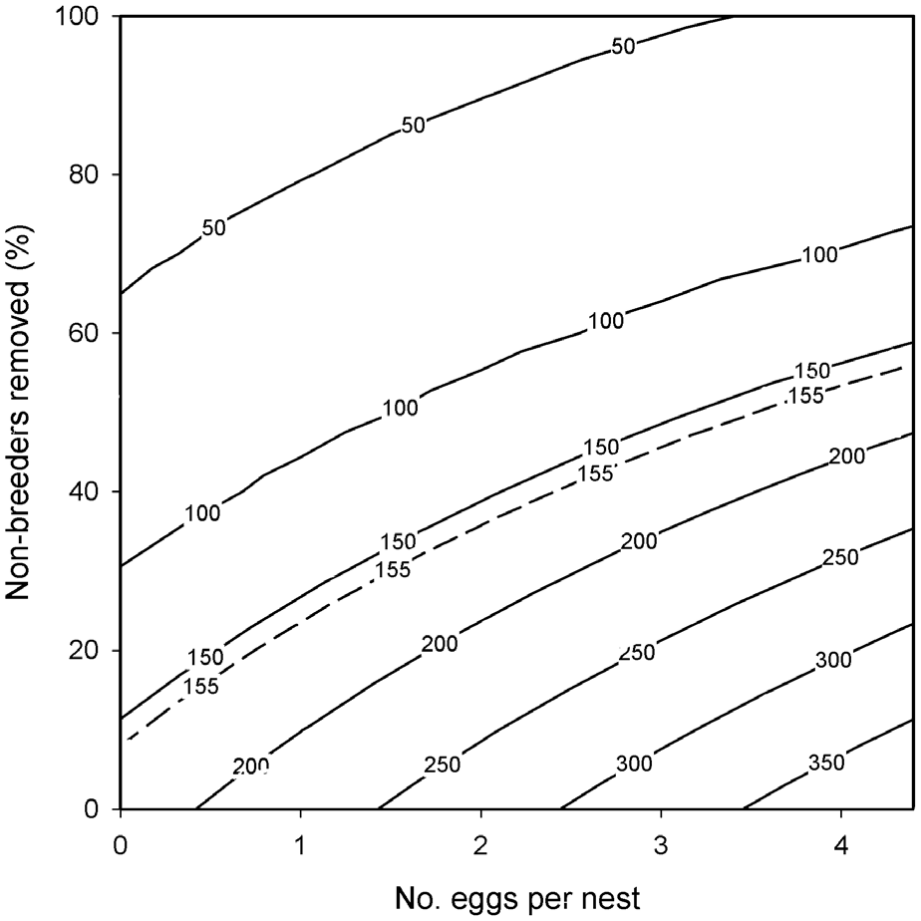
The projected stable population sizes, estimated as the mean population size between years 36 and 50, when subject to sustained population management over a 50 year period. Management comprised combined clutch manipulations (4, 3, 2, 1 or 0 eggs per nest) and the removal of non-breeding individuals (100, 80, 60, 40, 20 or 0%). The dashed line represents the target threshold of 155 individuals.

A range of different combinations of annual egg and non-breeder removals were projected to reduce population size below our target threshold of 155 swans (**Figure 4**). Typically, smaller reductions in clutch size and the numbers of non-breeders were required to achieve the management target when used in combination compared with either method used alone. For example, a reduction in clutch size to 2 eggs per nest, combined with the removal of 40% of non-breeding individuals, reduced population size to 146 swans in 16 years. Combined annual removals of all eggs and non-breeding individuals was projected to reduce population size from 257 to 31 swans in 3 years.

### Sensitivity Analysis

**Figure 5:**
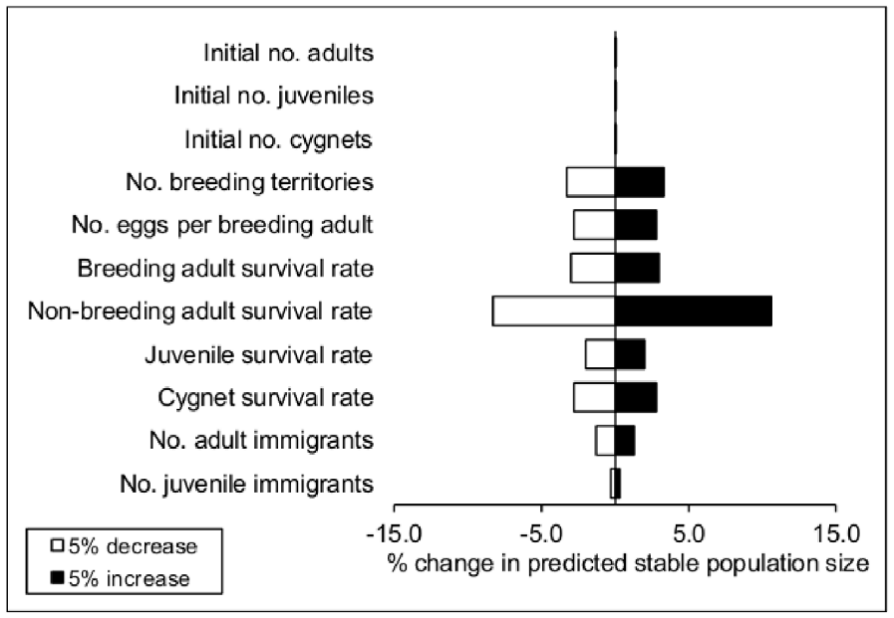
The sensitivity of the stable population size projected by the model to ±5% changes in the mean value of each parameter. We defined the stable population size as the mean population size between years 36 and 50 of the simulations.

Varying parameters by ±5% indicated that the stable population size projected by our model was affected most strongly by changes in the survival rate of non-breeding adults, which comprised the majority of the non-breeding population, with some, albeit lower, effects of the survival rates of breeding adults, juveniles and cygnets (**Figure 5**). The population size was not affected by altering the initial numbers of adults, juveniles and cygnets, with projected changes in population size of 0% for ±5% changes in the value of these parameters.

## Discussion

In this study we constructed and tested a mathematical model of mute swan populations causing conflicts with agricultural, fisheries and conservation interests. The model built and improved on previous swan population models by assessing management at the catchment-scale and by incorporating territoriality, which is a key factor regulating mute swan population dynamics [17], [36]. Thus our study represents the most comprehensive assessment to date of the potential of different methods of population management to alleviate swan grazing conflicts in chalk rivers. Our model accurately projected historical changes in the observed population sizes of two chalk rivers. We found that removing ≥60% of non-breeding swans each year, or management which combined egg removal and non-breeder removal, could reduce the swan population below the target threshold of 155 individuals necessary to prevent the grazing conflict. Grazing conflicts with large charismatic herbivores, such as ungulates and waterfowl, are increasing and effective management is required to alleviate ecological and socioeconomic damage [23], [24], [25]. Given the high costs and ethical issues associated with the implementation of population control measures, the arguments for or against a particular measure can be strengthened it is chances of success can be evaluated prior to field trials. This study has shown how mathematical population models can accurately project population trends, and be a useful method of evaluating the suitability of different management strategies.

In common with a range of other studies of animal populations, our model suggested that reductions in populations could be achieved through reduced productivity or the removal of individuals [4], [46], [47]. However, the size of these reductions were generally insufficient to alleviate the grazing conflict in our study system. Thus a projection of decreased population size does not necessarily mean that a conflict will be prevented. Therefore where population models are used to evaluate the efficacy of management techniques, target population sizes must be included. Such target population sizes should correspond to the population size below which the conflict will not occur. We found that removing ≥60% of non-breeding swans each year, or management which combined egg removal and non-breeder removal, could reduce the swan population below our target threshold of 155 individuals necessary to prevent the grazing conflict. This concurs with previous studies of herbivorous waterfowl which found that annual removals of <60% did not achieve sufficient population reductions to prevent grazing conflicts [12], [48], [49]. In contrast, reduced clutch sizes were projected to result in population sizes in excess of the target threshold, even where clutch sizes were reduced to 0 eggs per nest.

Several mechanisms were likely to have prevented clutch reductions and ≤60% removals of non-breeders from reducing population size below 155 individuals. Firstly, the high apparent survival rates of juveniles, non-breeding adults and breeding adults meant that these individuals persisted within the population. Similar studies have also concluded that adult survival rates have the greatest effect on model projections for long-lived herbivores [3], [50]. As high adult survival rates have a disproportionately large effect on population sizes, several studies have suggested that effective management should focus on adult mortality in preference to productivity [3], [51]. This perspective is supported by our finding that removing non-breeders, but not reducing productivity, could cause the required reduction in population size. Secondly, immigration of juveniles and non-breeding adults into the catchment partly offset population reductions due to management, which is known as the rescue effect in source-sink dynamics [52]. Although the annual number of immigrants was small relative to the total population size, such immigration prevented the extirpation of the population within our study area even when 100% of non-breeding individuals and all eggs were removed. Only ≥60% removal of non-breeders created a sufficiently strong population sink to overcome the rescue effect of immigration and thus allow population size to be reduced below the target threshold. Our results suggest that management of a small area, such as a single river catchment, is too limited for a highly mobile, widely distributed species such as the mute swan. These results concur with previous studies which concluded that population control would be ineffective where metapopulation dynamics of the target species, including the locations of population sources which produce individuals which can move to other areas, were not considered [16], [21]. Immigration of individuals from outside of the management area will at least partly offset removals due to management [16], [53]. Many of the successful uses of population control to reduce population sizes have been reported for small, isolated populations which are not subject to immigration [3], [45], [47], [54]. Therefore, whilst the catchment may be a useful management unit for wildlife managers, highly mobile animals such as swans are able to move freely between different river catchments [17], [36], [40], [55]. Thus for highly mobile species it may be more appropriate to consider population management at larger spatial scales, which consider metapopulation dynamics and incorporate the surrounding areas which provide the individuals which immigrate into the area of conflict (i.e. population sources). However, we currently lack sufficient quantitative data on swan metapopulation dynamics across multiple river catchments in the landscape, which precludes expanding the current population model. The variance associated with our parameters represented both sampling error and environmental stochasticity, thus it is unclear how key parameters such as survival and immigration might vary over time. The source of immigrants to river catchments in southern England is often unknown as birds arrive without identifying markers, rings or tags. However, of the known individuals most have travelled distances of <40 km from nearby river systems [16], [56], but individuals have arrived from France, Germany, Sweden and the Netherlands [55]. Additionally, as the grazing conflict is a localised, small-scale problem occurring at a small number of chalk river sites [28], [29], [30], it may be hard to justify large-scale management.

The annual removals of ≥60% of non-breeding individuals would require sustained, high-levels of management intervention in the swan population. Such removals could be achieved through licenced translocation or culling of individuals. Translocations would likely be the more ethically acceptable option, especially with the public, as it is non-lethal [12]. However, to be a viable management option translocation requires sufficient release sites and a low probability that the translocated animals will return [5]. The availability of enough suitable sites at which to release large numbers of swans (up to 123 individuals per year) is doubtful given the high mute swan population in Britain [31], [57]. Translocations also risk transferring the grazing conflict to other areas. The possible shortage of suitable sites, risk of conflict transfer, and possibility that swans may attempt to return to their original location, all suggest that translocation may not be a suitable management strategy. In contrast, culling would not face these three issues. However, culling would face widespread opposition from stakeholders, especially the public, ornithologists and some landowners [12], [14].

Due to the substantial ecological, practical and ethical obstacles to effective population control highlighted in our study, alternative management strategies for grazing conflicts such as habitat alterations or feeding deterrents could be explored. Currently, there are few published data on the efficacy of different habitat management strategies and other ethical interventions on alleviating the grazing conflicts associated with charismatic vertebrate herbivores. Further research should test whether changes in habitat management could alleviate grazing conflicts associated with herbivores. Economically or ecologically valuable plant stands and associated biota could be protected by the establishment of sacrificial feeding areas near to areas of overgrazing, as have been suggested for grazing conflicts between geese and agriculture [18], [58], [59]. Strategies which deter or prevent animals from feeding, such as scaring, repellents and fencing may also be used to prevent grazing damage by herbivorous animals [60]. Both sacrificial feeding areas and feeding deterrents have been used to successfully alleviate swan grazing conflicts with agriculture, and so may be effective in chalk river catchments [60], [61], [62].

Our results highlight the importance of considering the metapopulation dynamics of the target species. Management which focuses exclusively on a single population, or a limited spatial area containing only part of a population, may be confounded by immigration from other populations within the landscape. Given the limitations of population control, it is questionable whether such control should be attempted for well-established species subject to immigration and high survival [21]. Our study highlights the need to evaluate the population level effects of different management techniques, both alone and in combination, prior to field trials. Population models, such as the one presented here, can provide a cost-effective and ethical means of such evaluations.
